# Clinical Breast Examination As the Recommended Breast Cancer Screening Modality in a Rural Community in Malaysia; What Are the Factors That Could Enhance Its Uptake?

**DOI:** 10.1371/journal.pone.0106469

**Published:** 2014-09-04

**Authors:** Nik Daliana Nik Farid, Norlaili Abdul Aziz, Nabilla Al-Sadat, Mariam Jamaludin, Maznah Dahlui

**Affiliations:** 1 Department of Social and Preventive Medicine, Faculty of Medicine, University of Malaya, Kuala Lumpur, Malaysia; 2 Centre for Population Health, Department of Social and Preventive Medicine, Faculty of Medicine, University of Malaya, Kuala Lumpur, Malaysia; 3 Julius Centre, Department of Social and Preventive Medicine, Faculty of Medicine, University of Malaya, Kuala Lumpur, Malaysia; University of North Carolina School of Medicine, United States of America

## Abstract

Breast cancer is the most common cause of deaths and the most frequently diagnosed cancer among women worldwide. This study aimed to determine the prevalence of breast cancer screening, specifically on clinical breast examination, and the predictors of its uptake among women in Malaysia. A cross-sectional study was carried out in five selected districts whereby women aged between 20 to 64 years old, from a total of 1000 households were interviewed. A total of 1192 women responded to the survey of which 53.3% reported had ever done clinical breast examination. Significant associations with clinical breast examination were noted for income and distance from the hospital. These factors should be considered in developing interventions aimed at promoting clinical breast examination. In particular, healthcare providers should be proactive in raising awareness about clinical breast examination among women in Malaysia.

## Introduction

Breast cancer is the most frequently diagnosed cancer in women worldwide, with more than 1.5 million cases reported in 2012 [1]. Although the occurrence of breast cancer is higher in developed countries owing to demographic trends, its incidence is increasing in most African and Asian countries [2]. The incidence of breast cancer is escalating rapidly in Asia. In China and India, breast cancer rates have increased by up to 30% over the past 10 years, while in Japan, Korea, and Singapore, incidence rates have doubled or even tripled over the past few decades [3]. In Malaysia, breast cancer is the most commonly diagnosed cancer among women of all ethnic groups [4]. It accounted for 31% of newly diagnosed cancer cases in females [4]. The peak age of breast cancer presentations for Malaysian women is between 40 and 49 years of age compared with 50 and 59 years of age in the West [5].

The outcomes of breast cancer patients have been shown to be improved by clinical breast examination (CBE) [6]. This modality is an established method for diagnosing breast cancer and is used worldwide, including Malaysia. In places that lack screening mammography, CBE is one of the most important methods for breast cancer detection. The technique involves a thorough physical examination of the breast including visual inspection, checking for palpation of the breast, and examination of the axillary lymph nodes [7]. The technique detects more than 50% of the cancers seen on screening mammography and may improve breast cancer survival rates. Moreover, CBE identifies some cancers not found by mammography [7]. The technique may be of great significance for women who do not receive regular mammograms, either because mammography is not recommended or because they received mammography that is inconsistent with recommended guidelines [7]. Moreover, CBE’s input to women’s health may extend beyond its capability to identify previously undetected palpable masses [8]. All health workers should be encouraged to use the opportunity to conduct CBE whenever a woman presents themselves to the clinic or health care setting (opportunistic screening). Specifically, CBE presents an opportunity for health workers to guide women about breast cancer, its symptoms, risk factors, and development in early detection, as well as normal breast composition and variability. It lets clinicians discuss other screening methods such as breast self-examination (BSE), benefits and limits of BSE, and show BSE to women who prefer to do it. In addition to that, a CBE session provides opportunities for health education as well as improves the skills of health care providers.

Acknowledging the importance of early detection, several countries, including Malaysia, have adopted breast cancer screening programmes as part of their cancer control strategies. Breast Cancer Prevention Program Health Awareness organized by the Ministry of Health Malaysia promotes physical examination by healthcare professionals as part of their secondary prevention programmes [9]. It promotes annual CBE for women aged 40 years or more [9]. The screening policy for breast cancer focuses on CBE, whereby all women aged 20–39 years must undergo breast examination by trained healthcare providers once every 3 years and those aged 40 and above should get themselves examined annually [10]. Besides CBE, other screening modalities such as BSE and mammogram are also included in the program [10].

Results from clinical trials have shown no direct evidence that population-based screening using CBE is effective in reducing the number of breast cancer mortalities [11]. However, regular CBE may offer some benefit for women who are not participating in regular mammographic screening [11]. In Malaysia, the prevalence of mammographic screening uptake is low due to barriers such as embarrassment, presence of male technicians/radiographers and low confidence with radiologist/radiographers in detecting abnormality [12]. Furthermore, previous studies have shown no difference in breast cancer mortality between CBE and mammography [13]. Therefore, improving CBE by providing better training to nurses and doctors as well as promoting early detection of breast cancer by CBE could enhance this screening modality.

There are limited studies pertaining to prevalence and factors associated with CBE. Most studies have looked into mammography and BSE or breast screening in general. In Malaysia, The National Health Morbidity Survey in 2006 reported that the prevalence rate of CBE uptake was 51.8% [14]. In the United States of America, the prevalence of CBE among women in districts of Pennsylvania was compared between ages of 40–49, 50–64 and 64–65. The study showed that there is an inverse relationship between age and CBE [15]. The prevalence percentages decrease for older Pennsylvanian women. Among the three age categories, the 40–49 age groups had higher percentages which ranged between 89–94 percent [15].

Recognising the risk factors associated with breast screening uptake is important to improve the uptake and overall outcome of the disease. Previous studies have found that uptake of screening is associated with socioeconomic status and distance from the screening location [16]. Other factors that have been shown to influence screening uptake are ethnicity, education, and awareness of risk factors and screening methods [17]. With regards to CBE, the prevalence percentages increased with higher household income levels for Pennsylvanian women who have ever had a clinical breast exam. In one of the districts of Pennsylvania, The CBE percentages averaged around 96 percent for women with an income of $50,000 or more. For women with a household income of less than $10,000, the percentages ranged between 68 and 75 [18].

In Malaysia, the incidence of breast cancer and presentation at a later stage remains an issue [19]. Despite the availability of health education, facilities, and healthcare providers trained to conduct CBE in government health clinics, the awareness of CBE remains below the desired level [20]. Hence, this study aimed to determine the prevalence of CBE and the associated factors among women in Pahang.

## Materials and Methods

Ethical approval was obtained from the Ethical Committee of the University Malaya Medical Centre. Informed consent, both written and oral were obtained from all participants.

This is a cross sectional study involving rural communities within five selected districts in Pahang. The districts were determined by Pahang State Health Department. The study was conducted in February 2014. The data are drawn from the Community Residency Programme (CRP) Study which is done yearly in a state in Malaysia. The state of Pahang was chosen due to its representative nature of the whole of Malaysia as depicted by its population structure and composition. Located on the east coast, Pahang is the third largest state in Malaysia [21]. The population of Pahang was more than 1.4 million, with an average annual population growth rate of 0.5% [22]. The districts included in the study were Jerantut, Bentong, Raub, Temerloh, and Maran, centrally located in the state of Pahang. Four rural villages were selected from each district. These villages included Orang Asli or Malaysian indigenous peoples’ settlements. About 1000 households had been selected by proportionate random sampling and visited for face to face interview by trained medical students.

The variables in the CRP questionnaires included living conditions, health status, physical activity level, nutritional status, and measurement of body weight, height and blood pressure other than the socio-demographic characteristics and KAP of breast and cervical cancer screenings. The questions were answered by individuals according to age and gender. As for the KAP of breast cancer screening, female aged 20–64 years, resided in the village in the last one month and able to communicate were eligible to for the survey.

The KAP of breast cancer questionnaire consisted of three sections: (1) knowledge and perception of breast health (2) breast cancer screening practices, and (3) social support. Seven questions were used to assess the respondents’ knowledge of breast cancer. A scoring system based on previous local studies utilizing the same questionnaires was used to classify the level of knowledge in this study [23]. Correct answers were given one mark and incorrect answers were given zero mark. The marks obtained from these questions were totalled and classified into three levels of knowledge. A total score of 4–5 was considered as ‘good’, 2–3 as ‘moderate’, and 0–1 as ‘poor’ knowledge of breast cancer. A CBE is a breast examination by a health professional such as a doctor or nurse [24]. This variable was measured by asking respondents “When was the last time you had breast examination by a nurse?” Responses were “State in ___months ago” and “I have never undergone breast examination by a nurse”. Social support is a measure of the perception and reality of whether the women are cared for, have assistance from other people, and are part of a supportive social network [25]. These supportive resources can be emotional, physical, informational, and companionship-related. Ethical approval was obtained from the Ethical Committee of the University Malaya Medical Centre.

The collected data were analysed using the SPSS statistical software package version 22.0. Data from each district were analysed separately and merged for comparison. Chi-square tests were applied for comparing the categorical variables among the groups and to show associations. The level of significance was set at p<0.05. Multiple logistic regressions (backward likelihood) were applied for assessing the effects of multiple independent variables (determinants) on the value of the dependent variable (outcome), as well as to determine the associations. The final model is based on factors that have a significance level of p<0.05.

## Results

### Socio-demographic

All women aged 20–64 were interviewed (participation rate of 100%). In total, there were 1192 women aged 20–64 years who responded to the survey. The proportion of women aged 51 years and above was higher (38.3%) than women in other age groups. The majority were Malays (72.4%), while 8.5% were Orang Asli (Malaysian aborigines), married or partner (87.7%), had at least one child (95.6%), and most studied up to the secondary level (48.6%). Additionally, more than half of the respondents were currently housewives (62.9%). The socio-demographic characteristics of the respondents are listed in Table 1.

**Table 1:** Socio-demographic Characteristics of Respondents.

Characteristic		Frequency	Percentage
Age group	*20–29*	252	21.2
	*30–39*	224	18.8
	*40–49*	230	19.3
	*50–59*	326	27.4
	*60–69*	159	13.4
Ethnicity	*Malay*	863	72.4
	*Chinese*	103	8.6
	*Indian*	112	9.4
	*Orang Asli*	101	8.5
	*Others*	13	1.1
Education level	*Primary*	354	32.7
	*Secondary*	527	48.6
	*Tertiary*	203	18.7
Marital status	*Single*	144	12.3
	*Partner*	1031	87.7
Parity	*Nulliparous*	53	4.4
	*Parous*	1139	95.6
Occupational status	*Unemployed*	738	62.9
	*Employed*	435	37.1
Body mass index	*Underweight*	110	9.3
	*Normal weight*	377	32.0
	*Overweight*	368	31.3
	*Obese*	322	27.4
Ever smoke	*Yes*	56	5.0
	*No*	1067	95.0
On Oral Contraceptive Pill	*Yes*	150	13.4
	*No*	969	86.6

The majority of the study population owned some type of vehicle: 75.6% owned a car and 91% owned a motorbike. In terms of distance from a public healthcare facility, more than 50% of the respondents lived within 20 km from the hospital. Additionally, 95.5% of the respondent’s houses were located about 20 km from the clinic. In terms of family income, 30.3% of the population generated monthly incomes of between RM1000–1999, followed by 27.2% earning between RM501–999, and 16.1% earned less than RM500 which is also below the Malaysian poverty line. About 15.7% women earned more than RM3000/month (GDP per capita of Malaysians is USD 15, 568 a year, equivalent to RM 3,891/month).

### Level of breast cancer knowledge

Out of 1192, only 151 (12.7%) scored well in terms of knowledge on breast cancer. The majority of them i.e. 585 (49.1%) had an average score, while 456 (38.3%) of the respondents scored poorly. The levels of knowledge were compared among the five different districts. Maran had the highest percentage of women with good or moderate knowledge of breast cancer, with 23.1% and 59.1%, respectively. Meanwhile, 48% of the respondents from Bentong were found to have poor knowledge score.

### Breast Self-Examination and Mammogram

There were 62.8% of the respondents who practiced breast self-examination (BSE). Among the women who practiced BSE, 68.1% performed it at regular monthly intervals, 8.3% performed it once every 2 months and 23.6% performed it once every 3 months. Only 14.1% of respondents ever had a mammogram screening.

### Clinical Breast Examination

The overall CBE uptake in a life time among the respondents was 53.3%. The proportion of CBE uptake within ethnicity showed that the highest was among the Chinese (65.9%), followed by Indian (61.3%), Malay (50.7%) and Orang Asli (37.1%). The majority of the respondents that underwent CBE (62.6%) did so annually. In terms of the percentage of respondents that practiced CBE among the five different districts, Bentong was the highest (70.5%), followed by Raub (62.3%), Maran (55.0%), Jerantut (44.9%), and Temerloh (38.8%).

**Figure 1:**
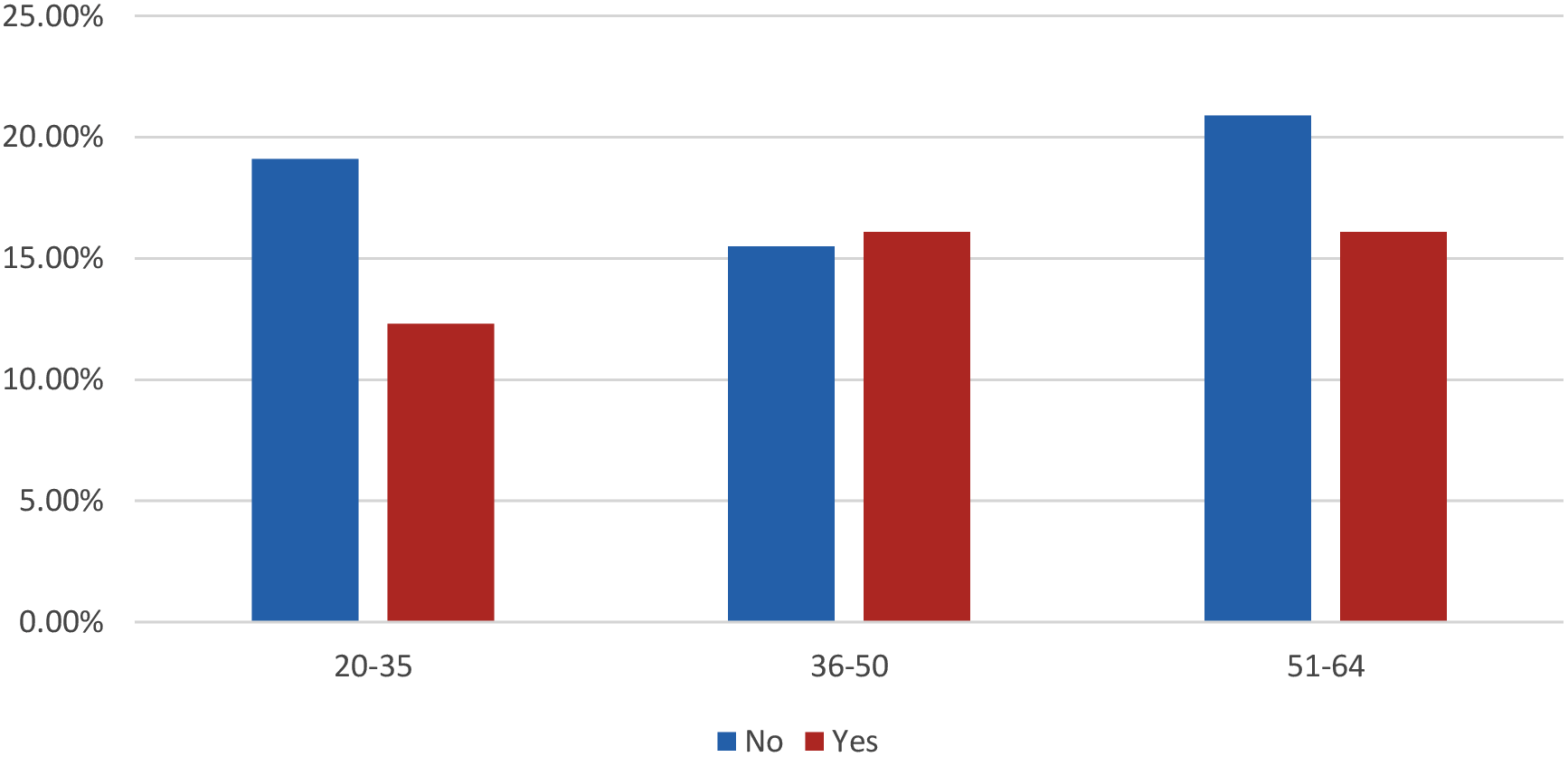
Last Clinical Breast Examination Conducted by Nurse.

The study showed that a majority of the women (98.9%) do not approve CBE to be performed by a male doctor. Out of the 674 women who had ever CBE, more than 61% had undergone CBE by a nurse, while less than 39% were examined by a doctor. Figure 1 shows the age group–based frequency distribution of women on whom CBE was performed by a nurse. Women in the 36–50 and the 51–64 age groups had the highest frequency (16.1%) of CBE by nurse.

**Figure 2:**
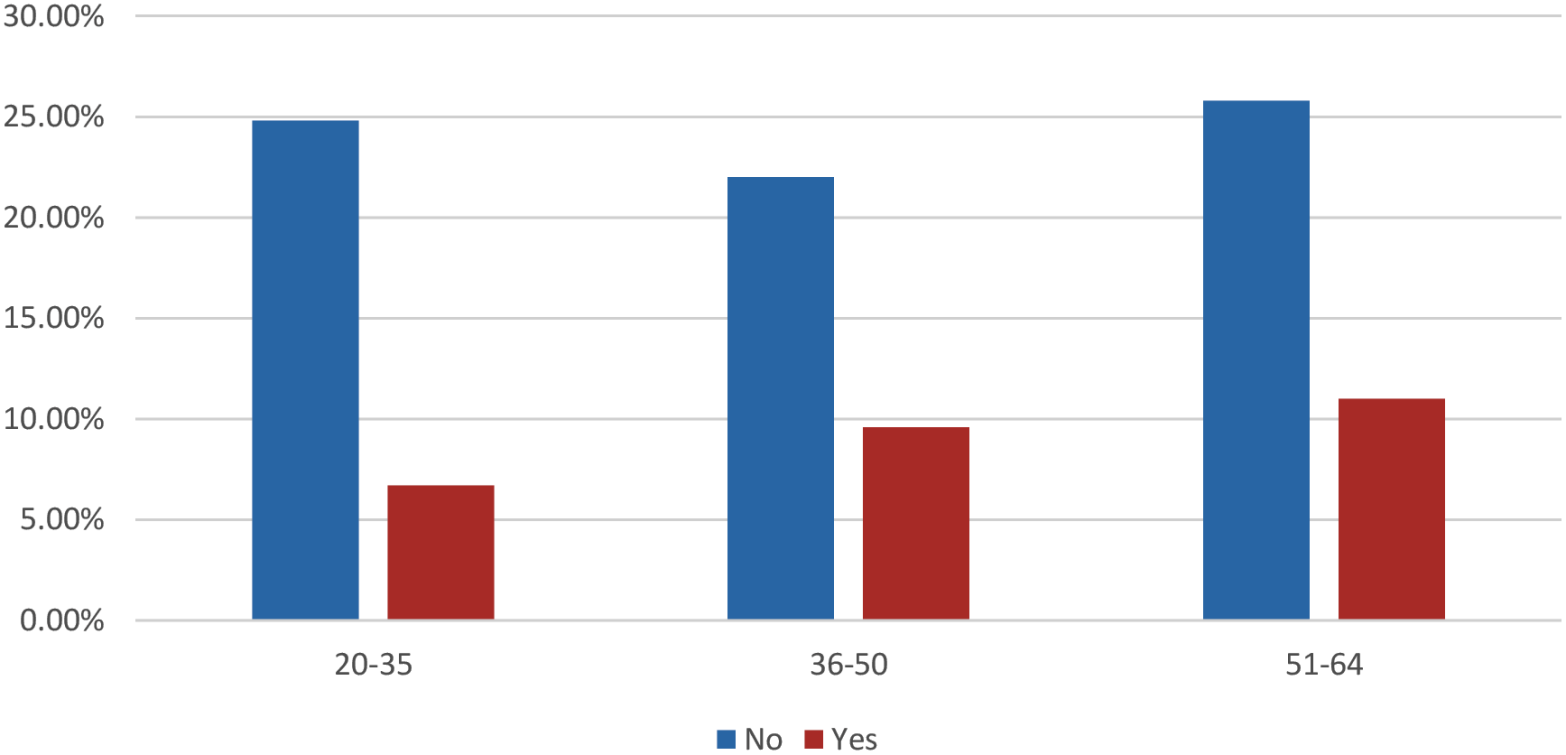
Last Clinical Breast Examination Conducted by a Doctor.

Figure 2 shows the age group–based frequency distribution of women on whom CBE was performed by a doctor. Women in the 51–64 age groups had the highest frequency (11.0%) of CBE by a doctor.

Table 2 shows the association between CBE and socio-demographic characteristics. There were significant associations between CBE and income (p<0.05) and distance from hospital (p<0.05). Women in the income bracket of RM 1000–1999 had the highest odds of undergoing CBE. In terms of distance from the hospital, those who lived within 20 km from the hospital were more likely to undergo CBE than those living farther away.

**Table 2:** Associations between socio-demographic characteristics and CBE.

Characteristics		Clinical Breast Examination		p-value	Odds ratio	95% CI
		Yes (%)	No (%)			
Income	*0–500*	11 (9.5)	22 (4.2)		1.0	(Ref)
	*501–999*	32 (27.6)	26 (28.6)	0.6	1.4	0.5–4.3
	*1000–1999*	37 (31.9)	21 (23.1)	0.01	4.8	1.4–16.2
	*2000–2999*	12 (10.3)	10 (11.0)	0.3	2.0	0.5–6.9
	*>3000*	24 (20.7)	12 (13.2)	0.2	2.3	0.7–7.7
Hospital within 20 km	*No*	208 (41.9)	227 (52.3)		1.0	(Ref)
	*Yes*	288 (58.1)	207 (47.7)	0.03	2.1	1.0–4.3
Education	*Primary*	136 (29.8)	132 (32.7)		1.0	(Ref)
	*Secondary*	242 (53.0)	177 (43.8)	0.07	1.33	0.97–1.81
	*Tertiary*	79 (17.3)	95 (23.5)	0.17	0.76	0.52–1.12
Occupational status	*Unemployed*	283 (59.0)	281 (65.0)		1.0	(Ref)
	*Employed*	197 (41.0)	151 (35.0)	0.06	1.36	0.99–1.70

Other factors found to be associated with CBE include having hormonal treatment (OR = 2.6, 95% CI: 1.1–5.8, p = 0.02), BMI of <18.5 (OR = 3.3, 95% CI: 1.1–9.9, p = 0.03), spousal support for breast cancer screening (OR = 1.8, 95% CI: 1.3–2.5, p = 0.001), and having a male doctor for breast examination (OR = 1.5, 95% CI: 1.1–2.1, p = 0.03).

Among the respondents, 46.7% did not practice CBE. Most of them stated that they did not perform breast examination screening because they practiced a healthy lifestyle (36.1%), did not have any such occurrence in their family history (10.9%), felt embarrassed (4.4%), were too busy (8.6%), and forgot their appointment (1.9%).

In terms of breast screening in general, 61.7% of the women received support from their spouse. More than 50% of the women refused to undergo breast examination by male doctors.

## Discussion

The purpose of this study was to determine CBE prevalence and investigate the factors that facilitate uptake or barriers to its uptake, among women in Pahang. From the survey, more than 53.3% of women aged 20–64 years reported that they had undergone CBE. The percentage is lower compared to the reported CBE uptake in other districts of Pahang and several rural districts of Perak [26]. However, the CBE prevalence rate in the current study is slightly higher than the 51.8% in the National Health and Morbidity Survey in 2006 [14]. In general the uptake of CBE among women of Malaysia was 50% to 60% which is fairly good but the periodic performance should be emphasized. A study in Kenya, Africa reported that less than 45.0% of women had ever undertaken CBE due to embarrassment and fear of breast cancer detection [27]. One likely explanation for the current study finding is related to poor awareness of CBE as a screening modality for good breast health. In addition, awareness campaigns on breast cancer and health may not be as extensive as in other places because of the distance and poor accessibility.

The Orang Asli or indigenous people suffer similar health problems as other ethnic groups. They represented nearly 10.0% of the women sampled in this study. However, only 37% had undergone CBE. This could be due to various factors such as cultural differences, language barriers, distance and limited awareness on breast cancer prevention. In addition to that, primary health care is still not available to all Orang Asli in Malaysia [28].

In this study, among women who underwent CBE, the majority (62.6%) reported being screened annually. This is because most women in this study were aged 40 years and above, and annual CBE is recommended for this age group.

In terms of practice of CBE among the five different districts, Bentong has the highest percentage of women who underwent CBE (70.5%) despite having poor knowledge on breast cancer. This could be ascribed to the proactive roles played by healthcare providers. Under the National Blue Ocean Strategy initiatives, nurses actively visit each house in their working territory to perform CBE as part of their routine.

More women had CBE performed by nurses than by doctors. This could be because of fewer doctors are posted in the rural areas of Pahang. Furthermore, owing to gender preferences, more women are comfortable and feel less embarrassed when examined by female nurses instead of male doctors. Also, it is because most of the women are seen by nurses at the Maternal and Child Health Clinic when they come for postnatal check-ups and family planning counselling.

This study showed that women in both the 36–50 and 51–64 age groups had the highest frequency of undergoing CBE by a nurse or doctor. This could be because more people within these age groups were captured in the study than those in 20–35 age groups. Furthermore, the older age groups (23.7%) tend to visit the clinic or hospital for medical problems compared to the younger age group (14.6%). Therefore, they are more likely to be aware of breast cancer and breast health programmes conducted in the primary care setting.

The results showed that there are two main factors (income and distance from the health centres) that significantly influence CBE uptake among women in the rural community. Women earning within the income range of RM 1000–1999 had the highest odds of undergoing CBE. Earning an income allows some female empowerment and thus better health practices. It is also noted that most of these women with income are able to afford to have their own transport and thus improves accessibility to a health care centre.

In terms of distance from the hospital, those who lived within 20 km from the hospital were more likely to undergo CBE than those living farther away from the hospital. For those living farther from healthcare facilities, having no transportation or living in area where public transport is limited makes it difficult for them to reach the hospital for screening. This finding is consistent with one study conducted in the United Kingdom (UK), whereby breast cancer screening uptake was higher among patients with a health facility located close to their area of residence [16]. Another study conducted among women in private households in the UK identified distance to a breast-screening unit as an important factor for screening [29]. In addition, this is because it is easier for them to access clinical facilities for CBE. Thus, the uptake of CBE could be enhanced if the barriers such as availability and accessibility of healthcare facilities can be addressed.

Although this study was only restricted to five districts in Pahang where most respondents were Malay, the results may represent the women of Pahang. This is because majority of people living in Pahang are Malays (50.4%) followed by Chinese (23.7%), Indigenous (11.0%) and Indian (7.1%) [30]. This study also has provided a brief description of the prevalence and determinants of CBE among the women in Pahang. Information bias is a limitation of this study. This is because of the respondents’ inability to recall the actual CBE that they underwent as well as other personal information. In order to limit this problem, each respondent was given time to recall and answer the questions.

## Conclusions

Breast cancer is a serious public health problem in Malaysia. It was found that higher knowledge score on CBE can contribute to early detection of some breast cancers and lead to prompt management and successful treatment of the disease. Thus, health planners must encourage the use of CBE as well as other screening modalities. Recognising the low uptake of mammogram in Malaysia, CBE remains the only option for early detection of breast cancer in Malaysia.

As distance is the main issue, especially, for women living farther from hospitals or healthcare centres, mobile units should be provided to increase CBE uptake. These mobile units should house nurses who are well trained in performing CBE. In addition, they should be able to educate people about and promote the importance of early consultation by breast surgeons on lump detection.

The Orang Asli community is scattered throughout Malaysia, with the largest population being in Pahang. Language and cultural factors should be considered when promoting CBE in the Orang Asli population. Since they have their own customs and beliefs, which may involve certain rituals and taboos, it is very important to make them aware of breast cancer and the available screening modalities. Doing so would empower the Orang Asli women and directly influence their decision-making process.

It is interesting to note that the highest CBE uptake is amongst those with lowest knowledge on breast cancer. This clearly showed the impact of healthcare intervention for instance rigorous campaigns conducted by health personnel. The Ministry of Health Malaysia’s plan to implement the concept of family doctor and strengthening of mobile services already have positive impact on CBE uptake as one health outcome.
